# Circulating Structurally Related (-)-Epicatechin Metabolite Species and Levels after Sustained Intake of a Cocoa Powder High in Polyphenols Are Comparable to Those Achieved after a Single Dose

**DOI:** 10.3390/nu13113829

**Published:** 2021-10-27

**Authors:** Paloma K. Barrera-Reyes, Josué Cortés-Fernández de Lara, Laure Poquet, Karine Redeuil, Martin Kussmann, Irma Silva-Zolezzi, Elizabeth M. Tejero

**Affiliations:** 1 Laboratorio de Nutrigenética y Nutrigenómica, Instituto Nacional de Medicina Genómica, Mexico City 14610, Mexico; pakabare@gmail.com (P.K.B.-R.); josuedan.cortes@gmail.com (J.C.-F.d.L.); 2 Universidad Nacional Autónoma de México, Mexico City 04510, Mexico; 3 Nestlé Research Lausanne, Société des Produits Nestlé, 57 route du Jorat, CH-1000 Lausanne 25, Switzerland; laure.poquet@rdls.nestle.com (L.P.); Karine.Meisser@rdls.nestle.com (K.R.); 4 Nuritas Limited, D02 RY95 Dublin, Ireland; Kussmann.martin@nuritas.com; 5 Nestlé Research, Société des Produits Nestlé, Singapore 618802, Singapore; Irma.Silva-Zolezzi@rdsg.nestle.com

**Keywords:** healthy adults, cocoa, polyphenols, epicatechin, epicatechin metabolites, quantification, antioxidant capacity

## Abstract

Background: While the bioavailability of cocoa polyphenols, particularly of the monomer (-)-epicatechin, has been investigated after a single-dose intake, the effect of sustained cocoa consumption on the metabolic profile of the structurally related (-)-epicatechin metabolites (SREMs) has not been investigated. Methods: A randomized, controlled crossover clinical trial in healthy young adults (18–40 year) was conducted to evaluate SREMs after consumption of a single-dose and after daily consumption of 1.3 g of polyphenol-rich cocoa powder for 28 days. The circulating SREMs were measured by ultra-high performance liquid chromatography-tandem mass spectrometry (UHPLC-MS/MS). Results: Twenty subjects (eleven males and nine females) were enrolled. The SREMs concentrations increased to 1741 ± 337 nM after a single-dose and to 1445 ± 270 nM after sustained supplementation. Sulfate conjugates showed higher levels in females (*p* < 0.05). The epicatechin-3′-glucuronide (E3′G) and epicatechin-3′-sulfate (E3′S) were the most abundant metabolites in all subjects. A high intra-individual correlation (r = 0.72, *p* < 0.001) between SREMs concentrations after single-dose and sustained supplementation was observed. The antioxidant capacity of plasma did not change in response to the intervention and was not correlated with any of the SREMs. Conclusion: The individual SREMs profile and concentrations after a 28-day supplementation are comparable to those after a single dose.

## 1. Introduction

Cocoa-derived products are widely consumed and appreciated thanks to their sensorial properties and high content of bioactive polyphenols [1,2]. The biological effects of cocoa polyphenols have been investigated and robust evidence supports their effects on cardiovascular [1,3] and immunological health [4,5] and, more recently, on the digestive system [6,7]. Cocoa polyphenols are found in monomeric (flavan-3-ols), and oligomeric or polymeric (proanthocyanidins, also named tannins) forms. The degree of polymerization influences the digestion and further metabolism of cocoa polyphenols, hindering the absorption of longer polymers. (-)-Epicatechin is the most abundant flavan-3-ol identified in cocoa, and oligomers and polymers may produce (-)-epicatechin via metabolism [8]. Recent data have shown that (-)-epicatechin, but not oligomeric and polymeric procyanidins, are responsible for the improvements in vascular function after cocoa intake [9]. However, its precise biological mechanisms of action remain under investigation.

Approximately 20% of (-)-epicatechin is absorbed in the small intestine by passive transport and is metabolized by phase II enzymes in enterocytes and hepatocytes [10]. Structurally related (-)-epicatechin metabolites (SREMs) have been identified in systemic circulation 15 min after consumption of a single-dose, mainly as glucuronide and sulfate conjugates [10,11]. These metabolites have a relatively short life in plasma, as they are cleared and eliminated via urine, and six hours after ingestion, their concentration in plasma is below detection limits [11,12]. After ingestion and absorption of the monomers and oligomeric forms, the remaining cocoa procyanidins reach the colon and interact with the gut microbiota to produce valerolactone and phenolic acids, named 5-carbon ring fission metabolites (5C-RFM) [13,14]. The second peak of 5C-RFM metabolites has been recently described in plasma after six hours of cocoa consumption [11]. The contribution of these metabolites to the systemic effects associated with cocoa intake has not been completely described, though it is believed to increase the population of specific bacteria and to impact the gut ecology.

According to previous studies [15,16,17], the circulating profile of SREMs is highly variable among subjects. Variability across different studies has been associated with individual characteristics, study design, and analytical differences. For instance, age, sex, and genetic background may influence the absorption and conjugation capacity of (-)-epicatechin and other polyphenols [18,19]. In addition, the vehicle used may affect the absorption rate and conjugation [20]. The time of sample collection after polyphenol intake and the analytical method may contribute to the reported variations in SREMs [18,20]. Different SREMs have been identified in animal and human studies after the intake of polyphenol-rich cocoa and chocolate. The most abundant SREMs found in human plasma include (-)-epicatechin-3′-glucuronide (E3′G), (-)-epicatechin-3′-sulfate (E3′S), and 3′-O-methyl-(-)-epicatechin-sulfate (3′MeES) [10,11,15,16].

The presence of these metabolites has been correlated with changes in the expression of human microRNA in in vitro studies [21], and their recovery in urine samples has been proposed as a reliable biomarker to estimate the (-)-epicatechin intake after a single-dose consumption [12]. Studies on other polyphenols have identified changes in the poly-phenolic metabolites in plasma and urine after 4 weeks of daily intake [22]. However, the effect of a sustained intake of high polyphenol-containing cocoa powder on the SREM profile has not been tested. Therefore, the present study aims to analyze the differences in the abundance and metabolic profile of SREMs in plasma after the intake of a single-dose or after sustained (28 days) consumption of high polyphenol-containing cocoa powder in young female and male adults.

## 2. Materials and Methods

### 2.1. Participants

Twenty healthy young adults (18–40 years) with a BMI between 18.5 to <30 kg/m^2^, non-smokers, and classified as sedentary to moderately active according to the International Physical Activity Questionnaire (IPAQ) [23] were recruited. Consumption of supplements, antioxidants, or medication one month before or during the study was an exclusion criterion. Participants were not allowed to modify their diet during the study and were asked to avoid polyphenol-rich products 24 h before sample collection. Subjects that did not fulfill the study criteria were not included in the analyses.

### 2.2. Study Design

The study was approved by the Ethics Committee of the National Institute of Genomic Medicine and was registered as a clinical trial at COFEPRIS (Federal Commission for the Protection against Sanitary Risk) according to Federal Regulations (registration number: 133300CT190199). All participants signed informed consent. The study was conducted at the Nutrition Clinic at Universidad Iberoamericana in Mexico City.

**Figure 1:**
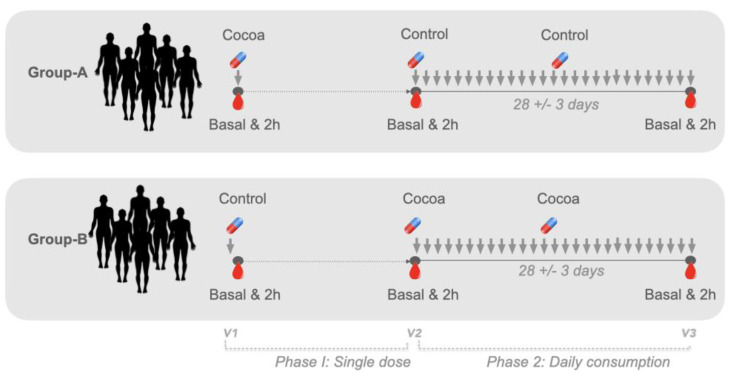
Study design. The study design was a double-blinded, randomized, crossover clinical trial for the evaluation of single-dose intake in healthy young adults (*n* = 10 per group) and followed by a controlled parallel-design for evaluation of sustained consumption consisting of daily doses of 1.3 g of the product for 28 ± 3 days (control *n* = 9 and cocoa *n* = 10).

The study consisted of two phases carried out in three visits. The first phase was a double-blinded, randomized, crossover study. At visit 1 (V1) and visit 2 (V2), the effect of the consumption of a single-dose of cocoa powder or control was assessed, with a one-week wash-out period between visits. Volunteers were appointed to the clinic and randomly allocated to Group A (cocoa–control sequence) and Group B (control–cocoa sequence). During V1 and V2, participants received a single dose of high polyphenol cocoa (1.3 g of test product) or maltodextrin. The volunteers were randomized by a third-party not directly involved in the study. After the cross-over study, the volunteers were assigned to a controlled parallel-design with sustained consumption of daily doses of 1.3 g of the product or the control (maltodextrin) for 28 ± 3 days (Figure 1). Visit-3 (V3) was appointed after 28 days of daily consumption of the studied product or control (Figure 1). Blinding was broken after data analysis.

### 2.3. Intervention and Data Collection

During each visit, two blood samples of 20 mL each were taken under the fasting condition (basal) and after 2 h of product ingestion. During each visit, the subjects were not allowed to eat any food in between sample collection and could only drink water. Food consumption was evaluated using a 24 h recall and a validated food frequency questionnaire (Sistema de Evaluación de Hábitos Nutricionales y Consumo de Nutrimentos; SNUT) [24]. The anthropometric measures (weight, height, and waist circumference) were conducted as described by Lohman et al. [25], and a body composition analysis (fat mass, fat-free mass, and body water) was also carried out at baseline using bioelectric impedance in In Body 250 equipment. Compliance with the treatment was assessed using daily records filled out by the subjects and by accounting for the number of pills returned at the end of the study. Participants with a compliance <80% were not included in the analyses.

### 2.4. Study Product

The cocoa pills contained ~1.3 g of a commercially available high polyphenol-containing cocoa powder containing polyphenols (Table 1), alkaloids (caffeine and theobromine), minerals, and other compounds. The placebo pills contained ~1.3 g of a partially hydrolyzed polysaccharide (maltodextrins). Both products were encapsulated and delivered in pills equal in appearance.

### 2.5. (−)-Epicatechin Metabolites Profiling

Briefly, metabolites were isolated from 6 mL of plasma collected in heparin-containing tubes (BD, 367884 Vacutainer ^®^, Franklin Lakes, NJ, USA). Sample collection was performed in the basal state (fasting) and 2 h after product consumption (Figure 1). (-)-Epicatechin and (+)-catechin were purchased from Extrasynthèse (Genay, France). SREMs standards, (+)-catechin-4′-*O*-glucuronide (C4′G), (+)-4′-*O*-methyl catechin (internal standard), (−)-epicatechin-3′-*O*-glucuronide (E3′G), (−)-epicatechin-4′-*O*-glucuronide (E4′G), (+)-3′-*O*-methyl epicatechin (3′ME), (−)-4′-*O*-methyl epicatechin (4′ME), and (−)-3′-O-methyl epicatechin-4′-sulfate (3′ME4S) were synthesized internally at Nestlé Research (Lausanne, Switzerland). (−)-epicatechin-3′-*O*-sulfate (E3′S) and (−)-epicatechin-4′-*O*-sulfate (E4′S) were purchased from Saint-Herblain, France). The internal standards umbelliferone sulfate and umbelliferone glucuronide were purchased from Toronto Research Chemical (Toronto, Canada) and Sigma Aldrich (Buchs, Switzerland), respectively.

(−)-Epicatechin metabolites from cocoa powder were identified and quantified in plasma samples using UHPLC–MS/MS operating in electrospray negative ionization mode (Waters Acquity hyphenated to an AB Sciex QTRAP5500), according to the method described by Actis-Goretta et al. [10]. Sample analysis was carried out in duplicates as previously described [15]. Internal standards were spiked in all plasma samples for accurate quantification. The samples were analyzed and processed using Analyst Software, version 1.6.

### 2.6. Quantification of Total Antioxidant Activity of Plasma

The total antioxidant capacity in plasma (ACP) was measured using the Antioxidant Assay Kit from Sigma-Aldrich (St. Louis, MO, USA) according to directions of the manufacturer. The total ACP was measured using calibration curves built with six standards concentrations (0, 0.015, 0.045, 0.105, 0.21, and 0.42 mM) and using TroloxTM (water-soluble vitamin E analog) as the standard or control antioxidant. All samples were analyzed in duplicate.

## 3. Results

**Figure 2:**
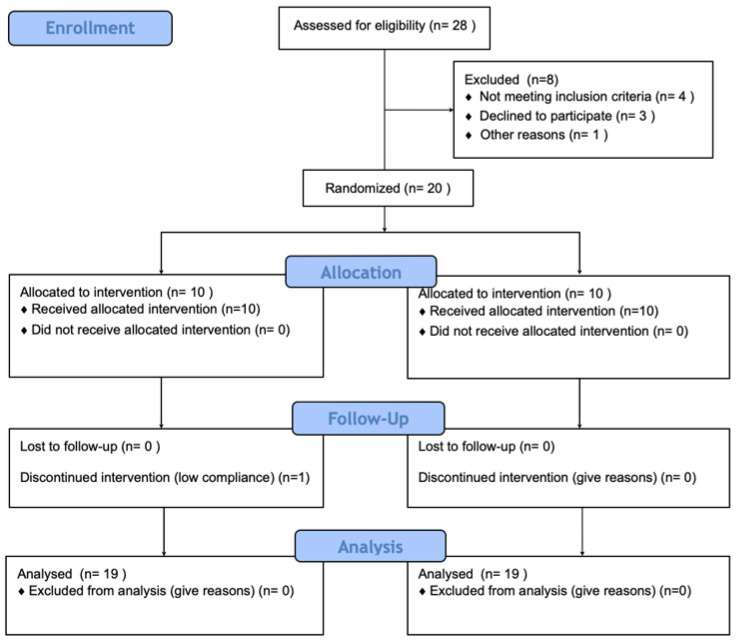
CONSORT statement. Participants allocated to Group A consumed the treatment in a cocoa–control sequence and those in Group B used the opposite order. Twenty participants completed the first phase of the study (single-dose), and nineteen completed the second phase (sustained consumption).

Twenty healthy young adults (nine females and eleven males) were enrolled in the study; all finished the first phase of the intervention (single-dose) and nineteen volunteers finished the second phase (sustained consumption for 28 days) (Figure 2). Half of the volunteers were randomly assigned to Group A (*n* = 10), and the other half were assigned to Group B. After unblinding, Group A was identified as the cocoa–control sequence, and Group B had the inverse intervention order. The data from the descriptive variables (anthropometry, physical activity, biochemical markers, and nutrition) were similarly distributed between groups at the basal state (Table 2). No adverse effects were reported during the study.

### 3.1. (-)-Epicatechin Metabolites

**Figure 3:**
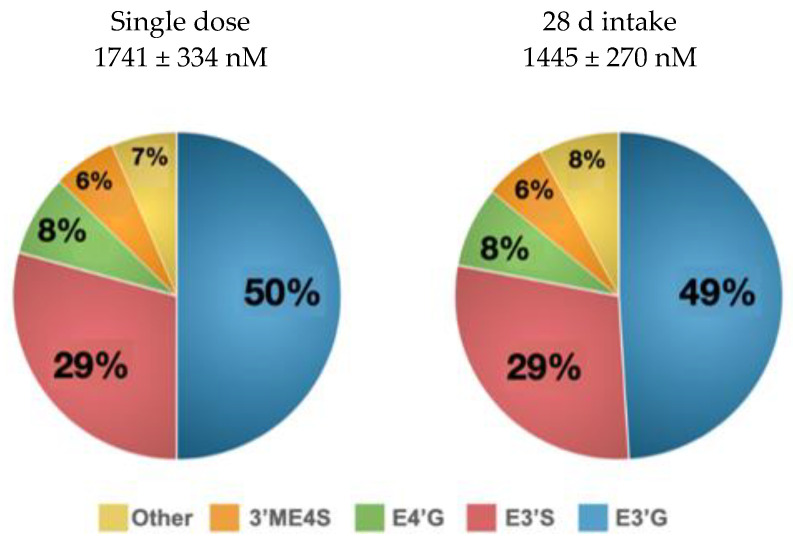
Structurally related (-)-epicatechin metabolites (SREMs) in plasma samples. Ten SREMs were quantified in the plasma samples by UHPLC-MS/MS. An analysis of the single dose was conducted at V1 and V2 (*n* = 20), and the 28 day consumption was analyzed at V3 (*n* = 10). The data are presented as the mean percentage after consumption.

None of the SREMs were identified in the plasma samples at baseline or after ingestion of the control treatment, indicating compliance with the inclusion criteria. After polyphenol-rich cocoa powder intake, seven of the ten metabolites increased their abundance with the exception of (+)-catechin, 4′ME, and 3′ME. No significant differences in the SREM concentrations or metabolite profiles were observed between the single and sustained cocoa consumptions for 28 days (Figure 3).

**Figure 4:**
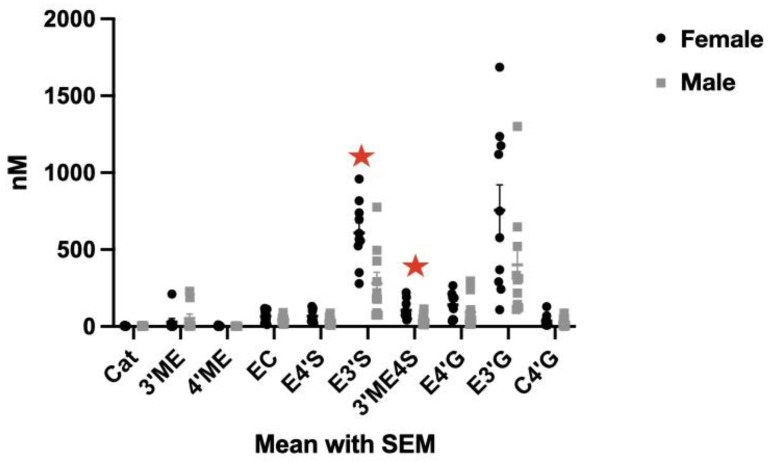
Abundance in the plasma of structurally related (-)-epicatechin (SREMs) metabolites by sex. An analysis of the single dose was conducted at V1 and V2 (*n* = 20)*. Catechin* (*Cat*), (+)-3′-*O*-methyl epicatechin (3′ME), (−)-4′-*O*-methyl epicatechin (4′ME), epicatechin (EC), (+)-4′-*O*-methyl catechin, (−)-epicatechin-4′-*O*-sulfate (E4’S), (−)-epicatechin-3′-*O*-sulfate (E3´S), (−)-3′-O-methyl epicatechin-4′-sulfate (3′ME4S), (−)-epicatechin-4′-*O*- glucuronide (E4′G), 3′-*O*-glucuronide (E3′G), and (+)-catechin-4′-*O*-glucuronide (C4′G).

The most abundant metabolites (E3′G, E3′S, E4′S, and 3′ME4S) accounted for ~85% of total SREM abundance. As observed in Figure 4, the increase in the abundance of SREM is different across individuals (high inter-individual variation); however, the response in the same individual remains relatively stable after either a single-dose or sustained intake. A strong intra-individual correlation; r = 0.72, *p* < 0.001) was observed in all participants. No differences by sex were noticed although the concentrations of E3′S and ME3′S were higher in female (*p* < 0.05). No carryover effects between visits or groups were found (*p* > 0.05).

### 3.2. Antioxidant Capacity

The change in the ACP was evaluated using the Trolox Equivalent AC method. In the present study, no significant differences were observed in the antioxidant capacity of plasma (ACP) after a single-dose or sustained consumption of cocoa or control treatments. Furthermore, the antioxidant capacity of plasma was not correlated with the concentration of any of the SREMs.

## 4. Discussion

The present study aimed to compare the bioavailability and SREM profiles of cocoa polyphenols after the intake of a single-dose and sustained consumption for 28 days of a high-polyphenols cocoa powder. The metabolic profile of SREMs and their possible association with changes in the antioxidant capacity of plasma were also investigated. Since the study considered a crossover design to evaluate the single-dose effect and a parallel design for sustained consumption, the inter-individual and intra-individual variability as well as the differences between treatments and supplementation periods were assessed. To the best of our knowledge, this is the first study that evaluates the influence of sustained supplementation with cocoa polyphenols on the abundance and metabolic profile of SREMs in healthy male and female participants.

The half-life of circulating SREMs is relatively short. This finding has been consistently reported [26,27,28]. The latest pharmacokinetic studies using radiolabeled [2-^14^C](-)-epicatechin suggested that SREMs are detected in circulation 15 min after the consumption of cocoa polyphenols and reaches a *C_max_* at 1–2 h and that their concentration falls below the detection limit at 8 h post-consumption [11]. In the present study, the SREM concentrations were below the detection limit in subjects under fasting conditions and these levels were not modified after the intake of control treatment. The baseline levels of SREMs increased after high-polyphenol containing cocoa consumption. Previous studies have reported circulating levels of SREMs in the range of 878–1520 nM, depending on the study design (dose, test product, and determination time) and analytical method (presence or absence of internal standards) [10,11,15,16,17]. In the present study, the abundance of SREMs after a single-dose intake was slightly higher than in previous publications (1741 ± 337 nM). This observation could be associated with several factors, including the basal and metabolic characteristics of the participants (fasting vs. post-meal), the vehicle used (dark chocolate vs. cocoa beverage vs. encapsulated cocoa powder), the time of measurement (1 h vs. 2 h), the doses (0.9–1.4 mg of (-)-EC/kg of body weight), and analytical differences such as the used chemical standards.

The SREM concentrations after sustained supplementation were similar to those observed after a single-dose intake. Slight reductions in inter-individual variability and mean abundance of SREMs were observed after 28 day of supplementation (1445 ± 270 nM), although the differences were not statistically significant. The results from daily supplementation suggest the following:

i. The production of phase II metabolites of (-)-epicatechin in the liver and small intestine remained relatively stable after repeated doses with no significant differences in the production of the biotransformed metabolites.
ii. The continuous excretion of SREMs does not allow for accumulation in the body, albeit biological effects have been observed after chronic interventions [2,29,30,31]; most of these studies have attributed the observed effects to SREMs. Recent data suggest, however, that cocoa polyphenols may also influence biological functions through different mechanisms, including the contribution of colonic metabolites [7].
iii. Long-term interventions using the same vehicles and doses may predispose to a more homogeneous response by reducing the initial variability across individuals, which is influenced by former dietary habits [32]. Future studies should evaluate the pharmacokinetics of SREMs at longer interventions, taking into consideration the effect of other colonic metabolites.

The identification of new SREMs has evolved in parallel with the development of more sophisticated methods for the identification and quantification of SREMs [14]. Ottaviani et al. and Actis-Goretta et al. were the first to quantify the abundance of SREMs using (-)-epicatechin metabolites [10,33]. They observed that E3′G and E3′S were the predominant SREMs in plasma, and their results were further confirmed. The findings from the present study are in line with previous observations and demonstrate the stability of the SREM profiles after sustained supplementation, even though the lack of a chemical standard for the measurement of 3′ME5′S did not allow for absolute quantification of this metabolite. Additional factors contributing to differences in the metabolic profile of SREMs across studies may be related to the cocoa product used and the ratio of stereoisomers ((-)-epicatechin and (+)-epicatechin) present in it, which may favor glucuronidation and sulfation at expenses of methylation during phase II metabolism [14]. Future investigations should provide a complete analysis of the stereoisomers present in the experimental vehicle to minimize this source of variation.

The bioavailability and pharmacokinetics of (-)-epicatechin are influenced by the study design (e.g., subjects characteristics, time for sample collection, administered doses, duration of the study, and analytical methods) [20,32]. Previous studies have quantified the SREMs in participants under fasting and non-fasting states, which influence the results due to the effect induced by other nutrients and their impact on the bioavailability of (-)-epicatechin [10,33]. The heterogeneity of the administered doses, which varies from 60 to 126 mg of (-)-epicatechin, could affect the conjugation profile during phase II metabolism; higher doses may promote conjugation with glucuronide groups at the expenses of sulfates [10,15].

The complexity of the food matrix used (e.g., isolated (-)-epicatechin, encapsulated cocoa powder, water-based and milk-based beverages, and chocolate bar) may elicit a differential gastrointestinal response depending on the nutrient content [32,34]. The time at which a *C_max_* is observed varies according to subject characteristics and the food matrix. Maximal concentrations have been identified 1 h post-consumption of cocoa drinks [11] or at 3 h post-consumption of dark chocolate in subjects who had breakfast [10]. This suggests that differences in the reported results were derived from the heterogeneity of the intervention. In the present study, an encapsulated polyphenol-rich cocoa powder containing 94 mg of (-)-epicatechin was administered to healthy subjects under fasting conditions. The simplicity of the food matrix and the subject’s characteristics minimized the metabolic or nutrient interactions, and other confounding effects. The administered dose is within the range of previous studies and is achievable by dietary consumption; therefore, the results of this study may reflect what happens in populations with high consumption of cocoa polyphenols.

High inter-individual variability was observed in the SREM profiles. Variability has been previously reported and linked to genetic and non-genetic factors such as age, body composition, sex, gut microbiota, and lifestyle, which could influence the enzymatic activity of the host [16,32,35]. Previous inter-individual variability has been estimated in the range of 38–37% (AUC, 0–6 h) after the consumption of cocoa flavanols [12,16]. A higher abundance of glucuronide conjugates was quantified in male subjects who are older (65–80 years) compared with those who are younger (18–35 years), although the total abundance did not differ between groups [16]. Scarce data regarding sex-dependent differences on the SREMs profile have been reported as most published investigations were carried out in males. In the present study, higher mean abundances of E3′S (610 nM vs. 281 nM; *p* < 0.05) and 3′ME4S (106 nM vs. 41 nM; *p* < 0.05) were found in females than in males, although non-significant differences in the total concentrations were observed between sex groups. A higher abundance of glucuronides has been previously reported in females after supplementation with resveratrol, possibly associated with sex-specific UDP-glucuronosyltransferase isoenzyme expression profiles regulated by sex hormones [19]. Future studies should address sources of inter-individual variation, including sex-dependent differences since the efficacy and biological effects of these and other bioactive molecules depend on their bioavailability and metabolism [18].

Low intra-individual variation in the abundance and SREMs profile was observed after exposure to a single-dose and 28-day supplementation with high polyphenol-content cocoa powder, suggesting that data from single-dose interventions may be reflective of the abundance and metabolic profile of SREMs in the longer-term. Similar results have been observed on circulating SREMs and urinary E3G, E3S, and 3′ME5S after the consumption of two single-doses of cocoa flavanols (CV = 12–13% and Pearson’s correlation coefficient: 0.83) [12,16] and after consumption of 400 mg of epicatechin gallate from green tea twice a day for four weeks [36]. The single-dose and daily consumption of blueberry polyphenols for 30 days also showed a modest increase in the plasma phenolic metabolites without differences in the total urinary excretion of polyphenols [37]. The sustained consumption of dietary polyphenols could have a differential effect on the abundance of metabolites derived from hepatic and microbial catabolism [38,39], as observed by Zhang et al. [22], after the consumption of red raspberry polyphenols for four weeks. Although the microbial metabolism of cocoa polyphenols was beyond the scope of the present study, future investigations should consider it. Feliciano et al. reported similar findings after the intake of wild blueberries for 30 day in healthy male participants [38].

In the present study, the consumption of a high-polyphenols cocoa powder did not influence the antioxidant capacity of plasma. The antioxidant activity of cocoa and other flavonoid-rich products has been controversial since different results have been reported across studies [40]. However, the latest literature suggests that the effect of these molecules may derive from an indirect mechanism of action [5,15,40,41]. This hypothesis is supported by (i) the low concentration of circulating metabolites after cocoa oral intake, (ii) the absence of oxidation products of (-)-epicatechin (ortho-quinones or quinone-related adducts) after the consumption of radiolabeled (−)-epicatechin, and (iii) the differential expressions of inflammatory cytokines and other molecules involved in redox balance post-consumption [11,15]. In the present study, analyses of plasma antioxidant activity were carried out after single-dose and sustained supplementation. The findings suggest that neither the identified SREMs nor the sum of them correlate with changes in the antioxidant capacity of plasma. Future studies should focus on intracellular signaling pathways differentially modulated by SREMs and 5C-RFM, and their association with the antioxidant and anti-inflammatory response. Furthermore, it must be considered that changes are likely dependent on the individual characteristics of participants and that not all subjects will respond in an even direction/magnitude to the same intervention. Then, the results should be analyzed in the context of responders and non-responders when possible [15].

The strengths of the present study are the direct measurement of circulating flavan-3-ols metabolites in plasma, the comparison between the effects of a single-dose and sustained intake for 28 day, and the inclusion of male and female participants. It is important to mention that the study design allowed for a comparison of the effects within each participant, at least for the single-dose effect. Among the limitations of the study is the lack of analysis of a larger number of metabolites, the use of a different compound as the control (maltodextrin) instead of a placebo, as well as the absence of sample collection at more time points after intake to analyze the absorption and metabolism of polyphenols during a longer period.

## 5. Conclusions

In conclusion, the abundance and profiles of SREMs after sustained supplementation with high-polyphenols cocoa powder for 28 days are comparable with those obtained after a single dose. The abundance of neither single SREMs nor the sum of them changed the antioxidant capacity of plasma, although their presence may influence the redox response by modulation of the intracellular signaling pathways. While high inter-individual variability was observed at the beginning of the study, it decreased after sustained supplementation, suggesting that individuals exposed to the same long-term treatment may elicit a similar response. However, the factors that will determine the subject’s response to a greater extent will depend on the individual characteristics. Longer interventions that evaluate not only SREMs but also 5C-RFM are recommended in future studies. These findings will contribute to elucidating the effects of chronic exposure to cocoa polyphenols and their impact on human health.

## Figures and Tables

**Table 1:** Composition of the high polyphenol-containing cocoa product.

Compound	Mg/g
Total polyphenol Content	500
Epicatechin ^a^	78
Flavan-3-ols ^b^	200
Flavanols	100
Theobromine	50

Values are in mg/g of cocoa powder. ^a^ Determined by Folin–Ciocalteu as catechin. ^b^ Determined as catechin, epicatechin, B1, and B2.

**Table 2:** Descriptive characteristics of the participants at baseline. Mean (SD).

Variable	Group A *n* = 10	Group B *n* = 10	*p* Value *
Sex (male) (%)	4 (36%)	6 (67%)	
Age (years)	27.4 (4.0)	26.7 (5.2)	0.68
BMI (kg/m^2^)	23.7 (2.9)	23.0 (3.1)	0.65
Physical activity (METs)	2512 (2449)	2197 (1203)	0.42
Glucose (mg/dL)	90.9 (6.6)	90.5 (6.5)	0.88
Cholesterol (mg/dL)	158.4 (37.6)	189.8 (37.8)	0.06
HDL (mg/dL)	52.2 (13.0)	47.6 (12.3)	0.99
LDL (mg/dL)	90.2 (34.0)	117.5(34.7)	0.13
Triglycerides (mg/dL)	80.2 (27.2)	120.7 (56.4)	0.13
Energy intake (kcal)	1814 (518)	2103 (807)	0.29
Fat mass (%)	23.5 (5.7)	25.5 (5.7)	0.27
Lean mass (kg)	31.2 (9.5)	33.2 (12.6)	0.67

* *t*-test for independent samples.
